# Quercetin in semen extender improves frozen-thawed spermatozoa quality and *in-vivo* fertility in crossbred Kamori goats

**DOI:** 10.3389/fvets.2024.1385642

**Published:** 2024-05-13

**Authors:** Iqra Batool, Muhammad Hammad Fayyaz, Amjad Hameed, Syed Murtaza Hassan Andrabi, Rehana Kausar, Muhammad Shahzad, Yasin Mubashir, Ali Dogan Omur, Ghulam Murtaza, Allah Ditta, Tarique Hussain

**Affiliations:** ^1^Animal Sciences Division, Nuclear Institute for Agriculture and Biology College, Pakistan Institute of Engineering and Applied Sciences (NIAB-C, PIEAS), Faisalabad, Pakistan; ^2^Animal Sciences Institute, National Agricultural Research Centre, Islamabad, Pakistan; ^3^Nuclear Institute for Agriculture and Biology College, Pakistan Institute of Engineering and Applied Sciences (NIAB-C, PIEAS), Faisalabad, Pakistan; ^4^Department of Reproduction and Artificial Insemination, Faculty of Veterinary Medicine, Ataturk University, Erzurum, Türkiye; ^5^Livestock and Fisheries Department, Government of Sindh, Karachi, Pakistan

**Keywords:** quercetin, antioxidants/oxidants, spermatozoa quality and *in-vivo* fertility, CASA parameters, crossbred Kamori goats

## Abstract

This study investigated the antioxidant effect of quercetin-treated semen on frozen–thawed spermatozoa quality and *in-vivo* fertility in crossbred Kamori goats. In total, 32 ejaculates from four fertile bucks were diluted in Tris-based egg yolk extender with varying levels of quercetin (0, 1, 5, 10, and 15 μM). Qualified semen samples were pooled and frozen in French straws. The results revealed that the addition of quercetin in the semen extender increased (*p* < 0.05) frozen–thawed sperm total motility (TM), progressive motility (PM), rapid velocity (RV), average path velocity (VAP), straight line velocity (VSL), curvilinear velocity (VCL), and amplitude of lateral head (ALH) displacement in contrast to the control group. Quercetin supplementation had no effect on beat cross frequency (BCF), straightness (STR), and linearity (LIN) (*p* > 0.05). Quercetin showed significantly higher (*p* < 0.05) plasma membrane and acrosome integrity and viability (*p* < 0.05) of spermatozoa in contrast to the control group. Quercetin in the semen extender significantly increased (*p* < 0.05) superoxide dismutase (SOD), catalase (CAT), peroxidase (POD), ascorbate peroxidase (APX), and total antioxidant capacity (TAC) levels while reduced (*p* < 0.05) the contents of total oxidant status (TOS) and malondialdehyde (MDA), which were in contrast to the control group. Ultrasound results revealed that 24 out of 30 (80%) goats were found pregnant when semen was treated with 5 μM quercetin while the control group showed 18 out of 30 (60%) animals were pregnant. Thus, the study concluded that 5 μM quercetin-treated semen was found to be efficient, showed increased antioxidant status, and reduced oxidant production, leading to improved spermatozoa quality and *in-vivo* fertility in goats.

## Introduction

Artificial insemination (AI) is a reproductive biotechnology tool used worldwide for improving animal production. However, it is not being practiced in small ruminants in Pakistan due to factors such as the lower conception rate, the unavailability of goat semen in the market, and a lack of awareness of AI (1, 2). The reluctance to adopt AI is because of the semen cryopreservation process, which reduces sperm viability due to the oxidative attack, resulting in a poor conception rate (3). Oxidative stress can cause deleterious effects on sperm concentration, sperm motility, sperm morphology, and sperm DNA damage, eventually resulting in apoptosis (4, 5). The sperm plasma membrane is a rich source of lipids and is more vulnerable to lipid peroxidation (6). The sperm cells consist of both enzymatic and non-enzymatic antioxidant enzymes, which aid them to protect against oxidative damage. Depleted antioxidant capacity is a presumed indicator of sperm infertility, resulting in a lower conception rate in animals (7). The addition of antioxidants or cryoprotectant media in semen extender improves sperm viability and the motility of fresh and cryopreserved ram spermatozoa (7, 8). The antioxidant capacity of frozen–thawed semen is not sufficient to overcome oxidative damage during the cryopreservation process. However, semen cryopreservation delivers successful results but declines sperm motility by 50% (9).

The cryopreservation process induces temperature alteration in spermatozoa, which makes them vulnerable to cryoinjuries such as ice crystal formation, oxidative stress, and cold shock (10–12). Egg yolks, which help prevent cold shock, are an essential energy source for semen extenders (13). However, the low-freezing potential of buck semen influences the pregnancy rate of artificial insemination (14). Buck sperms have been reported to be vulnerable to cryo-damages due to changes in seminal plasma across different species (15). The presence of phospholipase A causes the breakdown of fatty acids in buck seminal plasma, resulting in sperm lipid peroxidation (16) and the production of oxidants (17). Hence, centrifugation can be performed to eliminate the detrimental effects of seminal plasma interaction with egg yolks (18).

Quercetin is a flavonoid compound, which is well-known for its antioxidant properties, and is present in fruits and vegetables (19). Quercetin is a potent antioxidant that enables scavenging ROS and hydroxyl radicals (19). Moreover, quercetin has exhibited protective effects on fresh and frozen–thawed sperm parameters in diverse species (20–23) and is believed to be involved with α-tocopherol to delay oxidation and activate gene expressions of detoxifying enzymes (24). Antioxidants play a crucial role in the protection of spermatozoa from damage caused by oxidants, bacteria, leukocytes, and abnormal oxidants produced by sperm (25). Hence, cryoprotective media are subjected to be included in different antioxidants to optimize sperm cryo-survival (26, 27). Therefore, antioxidants, such as those with non-enzymatic and scavenging activities, are routinely employed in cryo-protective media (28, 29). The addition of quercetin to the semen extender provides a beneficial antioxidant effect on post-thawed semen attributes in rams (30) and bucks (31).

The application of estrus synchronization protocols during the on/off seasons improves fertility and allows kidding in goats at the desired period of the year (32, 33). In Pakistan, goats are bred through natural breeding due to the unavailability of goat semen units. Furthermore, AI services utilizing superior bucks are also negligible in government farms (34). The short and long-term protocols of estrus synchronization have been employed in cycling and non-cycling goats (35) with 40–80% fertility rates. Our locally developed polyurethane-based medroxyprogesterone acetate (MAP) sponges are cost-effective and offer precise estrus synchronization. Our previous studies on MAP sponges have exhibited promising results of MAP sponges in buffaloes, goats, and sheep (36–38). This study was designed to investigate the different levels of quercetin in semen extender on frozen–thawed spermatozoa quality and *in-vivo* fertility in crossbred Kamori goats.

## Materials and methods

### Ethical statement

The experiment was conducted following the guidelines of the Institutional Animal Care and Use Committee of the Animal Sciences Division, Nuclear Institute for Agriculture and Biology, Faisalabad, Pakistan, under the number NIAB-C-PIEAS-0023.

### Chemicals

The chemicals used in this study were procured from Sigma-Aldrich Chemical Co., USA and Merck, Darmstadt, Germany.

### Animal selection and management

The study was carried out at an animal Farm in the Nuclear Institute for Agriculture and Biology (NIAB), located nearly 7 Km from the center of Faisalabad city, Pakistan (longitude 73.0791° E and latitude 31.4287° N) with an altitude of 184 m above the sea level. The average temperature and rainfall were 26°C and 19.66 mm, respectively.

Four adult, fertile, healthy bucks of the Kamori breed, aged 8–12 months, weighing about 38 ± 0.8 kg were selected from Nawabshah, Sanghar, and Matiari districts of Sindh province, Pakistan. After purchasing, fertile bucks were kept in quarantine for 30 days to acclimatize and potentially identify any underlying diseases. Bucks were provided free grazing pasture for a period of 3–5 h/day, and green fodder was mixed with wheat bran, berseem, and concentrates (barley, wheat, oat, sorghum, rice, wheat bran, polished rice, molasses, and soybean meal) and supplemented at 250 grams/buck based on their body weight. Proper vaccination and deworming were done before the initiation of the experiment. Animals had *ad libitum* access to water.

### Semen collection, dilution, and freezing

Before the initiation of the experiment, bucks were properly trained with a teaser, and semen collection was carried out at an animal farm in the Nuclear Institute for Agriculture and Biology (NIAB), Faisalabad, Pakistan. Ejaculations (*n* = 32) from four active bucks (8 ejaculations/buck) were obtained through a caprine artificial vagina (IMV Technologies, France) during October and November 2023. Semen collection was performed every week for consecutive 4 weeks. After collection, the semen was brought to the laboratory and maintained at 37°C before evaluation. The motility (%) was assessed under a phase-contrast microscope (CX43, Olympus, Japan) at a magnification of X 40. The semen samples that had 3–4 × 10^9^ sperm/ml determined through the Neubauer chamber (Germany) and showed greater than 75% motility were processed for further experimentation. The centrifugation was performed at 315 *g* for 3 min for the removal of seminal plasma (39). Thereafter, semen was divided into five aliquots and diluted with a Tris-egg yolk extender composed of tris of 3.07 g, citric acid of 1.64 g, fructose of 1.26 g, streptomycin of 100 mg, fresh egg yolk of 15% and glycerol of 5% to make the final volume of 100 mL, pH 6.8–7.2, and 425 mOsm (40). Then different levels of quercetin (0, 1, 5, 10, and 15 μM) were used in an extender to make five aliquots of semen conceded in a single step at 37°C and adjusted up to 50 × 10^6^ motile sperm/0.5 mL. The sperm concentration was evaluated using a hemocytometer (Neubauer chamber, Germany). Consequently, antioxidant-treated diluted semen was cooled at 5°C. The semen was aspirated into 0.5 mL French straws, sealed with straw sealer, and then equilibrated at 5°C for 4 h. Following equilibration, the semen straws were frozen in liquid nitrogen vapor (4 cm above the liquid nitrogen) for 15 min and then kept in liquid nitrogen for storage. Frozen semen was thawed using a digital thawing jug at 37°C for 30 s for freeze–thaw evaluation.

### Freeze–thaw sperm evaluation

#### Sperm kinematics analysis

Cell Motion Analyzer (CEROS, Hamilton Thorne Biosciences, Beverly, USA) was used to evaluate the characteristics of sperm motion. The CASA system was programmed to the following settings: frames acquired, 30; frame rate, 60 Hz; minimum contrast, 56; minimum cell size, 5 pixels; minimum static contrast, 30; straightness threshold, 80%; average path velocity cutoff, 20 μm/s; progressive minimum average path velocity, 80 μm/s; curvilinear velocity cutoff, 0 μm/s; cell size, 2 pixels; cell intensity, 50; static head size, 0.71–10.00, and static head intensity, 0.79–1.41. A preheated slide (37°C) was loaded with 5 μL of semen sample and microscopic fields with the best visual motility. The microscopic image showed at least a count of 200 spermatozoa under magnification of × 910 in two to five fields. The motility parameters were the percentage (%) of motile and progressive motile spermatozoa. Velocity parameters of average path velocity (1 μm/s), straight line velocity (straight line velocity; 1 μm/s), and curvilinear velocity (1 μm/s) and frequency parameters of the amplitude of lateral head displacement (μm), beat cross frequency (Hz), straightness (STR, %), and linearity (LIN, %) were recorded. A description of these parameters of CASA was described in the previous publication (41).

#### Functional plasma membrane integrity (PMI)

The sperm-vital plasma membrane integrity was analyzed following the protocol of Khalil Ur Rehman et al. (42). Approximately, 200 spermatozoa were measured using a phase-contrast microscope (magnification of x 400; BH2 Olympus, Japan). The intact spermatozoa had a white head and coiled tails; however, stained spermatozoa had uncoiled tails and showed a pinkish appearance in the sperm head.

#### Acrosomal integrity

Trypan blue-Geimsa staining assay was conducted to examine the sperm viability and acrosome integrity (43, 44). Nearly, 200 spermatozoa per sample were examined under a phase-contrast microscope (magnification, x 400). The spermatozoa with an un-stained head and the purple acrosomal region for viable with intact acrosome and the blue head region and the pale lavender acrosomal region for non-viable and non-intact acrosome were observed.

### Viability

A 5 μL of frozen–thawed semen samples was employed and mixed with 5 μL of eosin-nigrosin stain using a micropipette tip. A smear was made, air dried, and examined by using a phase-contrast microscope with X 40 (CX 43, Olympus, Japan) magnification. The image showed that sperms with pink heads were dead, and sperms with white heads were alive.

#### DNA integrity (DI)

The DI was determined by the Acridine Orange (AO) assay. Briefly, a smear of 100 μL of semen sample was prepared using a clean glass slide and air dried. The smear was submersed in fresh Carnoy’s solution (methanol and glacial acetic acid in a 3:1 ratio) for 2 h. After the fixation, the dried smear was incubated in tampon solution (0.1 M of citric acid and 0.3 M of Na2HPO4 were added in the ratio of 16:1; pH 2.5) for 7 min at 60°C. Furthermore, the smear was rinsed by using water and stained (in the dark) with AO stain (1,000 μg/mL stock solution using distilled water; Sigma Aldrich, St. Louis, MO, USA) for 5 min. Then the slide was rinsed, and the coverslip was placed on the wet slide. The stained slides were evaluated immediately under a fluorescent microscope (magnification × 400; Nikon Optiphot, Japan; 480/550 nm excitation/barrier filter) and with an excitation filter (460–570 nm) and an emission filter (460–610 nm). Each microscopic field was observed for a maximum period of 25 s. Approximately, 350 sperm per smear were examined for DNA integrity. Sperm having intact DNA emitted green fluorescence, while fragmented DNA emitted yellowish-orange-red fluorescence.

### The detection of antioxidant/oxidant enzymes

Four straws per group, each having 0.5 mL French straws (50 × 10^6^ sperm concentration), were subjected to thawing at 37°C for 30 s and centrifuged at 1000 x *g* at 4°C for 10 min. The pellet was centrifuged again at 1000 x *g* for 10 min after being washed in 50 mM potassium phosphate buffer (pH 7.0) to a concentration of 1 × 10^8^ spermatozoa/ml. Then, samples were sonicated at a 15 s interval for 2 min (Elma E 30 (H), D-78224 Singen, Germany) and subjected to the following parameters.

#### Superoxide dismutase

The assay solution for the inhibition of nitroblue tetrazolium (NBT) consists of 50 mM phosphate buffer (45) of pH 7.8, 13 mM of L-methionine, 57 μM of NBT, 0.004% of riboflavin, 0.025% of triton, and 50 μL of sample to make up the volume of 3 mL. The photoreaction was conceded out under the 15 W and the absorbance was recorded at 560 nm in the spectrophotometer. SOD activity was measured in one unit as the amount of enzyme required to inhibit a 50% reduction of NBT.

#### Peroxidase activity

The guaiacol peroxidase (POD) measurement was conducted using sperm cells homogenized in a 50 mM potassium phosphate buffer with pH 7.0, 0.1 mM of ethylenediaminetetraacetic acid (EDTA), and 1 mM of dithiothreitol (DTT). The POD activity was estimated using the protocol of Hameed et al. (46) with minor modifications. The 3 mL solution consisted of 50 mM phosphate buffer (7.0 pH, 20 mM of guaiacol, 40 mM of H2O2, and 0.1 mL of enzyme extract). Then, the reaction was initiated by the inclusion of enzyme extract. Enhanced absorbance of the reaction mixture at 470 nm was measured every 20 s for 1 min. The POD activity in one unit was referred to as an absorbance change of 0.01 units/min.

#### Catalase

The catalase activity of samples was estimated using 50 mM potassium phosphate buffer (pH 7.0) and dithiothreitol (1 mM) by referring to earlier protocols (47). The reaction mixture comprised 59 mM of H2O2, 50 mM of phosphate buffer (pH 7), and 100 μL of sample. The reduction pattern of absorbance was assessed every 20 s for 1 min at 240 nm and a unit of CAT activity was described as a change in absorbance in 0.01 min.

#### Malondialdehyde (MDA)

The MDA levels in semen samples were estimated calorimetrically using MDA as standard (48). The sample (25 μL) was homogenized in 0.1% trichloroacetic acid and centrifuged for 5 min at 14,000 × *g*. Then, trichloroacetic acid (20%) consisting of thiobarbituric acid (0.05%) was added to 1 mL aliquot of supernatants and heated for 30 min in a water bath. The reaction mixture was cooled and centrifuged for 10 min at 14,000 × *g*. The absorbance of supernatants was computed at 535 nm, and the value of non-specific absorbance (600 nm) was subtracted from it. The MDA contents were measured by a coefficient of extinction of 155/mM/cm.

#### Total oxidant status

The procedure of evaluating total oxidant status (TOS) is based on the oxidation of iron and iron complex in an acidic medium (48). Ferric ions interact with xylenol orange and form a particular colored complex. The intensity of the color is linked with the quantity of the oxidant molecule and can be measured using a spectrophotometer. The mixture for the analysis of TOS consisted of reagent xylenol (0.38 g in 500 μL of 25 Mm H2SO4), o-dianisidine 0.0317 g, ferrous ammonium sulfate 0.0196 g, NaCl 0.4 g, and glycerol 500 μL) mixed in sample. The absorbance was recorded at 520 nm after 5 min. The standards used were 5, 10, 15, 20, 25, and 30 μM/mL hydrogen peroxide equivalents. The calibration curve of H_2_O_2_ was used to calculate TOS and was denoted in μmol H_2_O_2_ equivalent mL^−1^.

#### Total antioxidant capacity (TAC)

The TAC samples were measured based on the reduction of a blue radical cation (2,2′-azino-bis-(3-ethylbenzothiazoline-6-sulfonic) (ABTS∙+) to its original colorless form ABTS by antioxidants (49). The solution to detect TAC consisted of reagent R1 (CH3COONa buffer and glacial acetic acid), reagent R2 (H2O2, Na3PO4 buffer, glacial acetic acid, and ABTS), and the sample. The reacted solution was incubated for 6 min at room temperature, and the absorbance was taken at 734 nm. The standards used were 0, 0.075, 0.125, 0.25, 0.5, 0.75, 1, 1.25, 1.5, 1.75, and 2.0). The TAC was calculated using ascorbic acid as a calibration curve and represented as ascorbic acid (μM) equivalent per milliliter of sample.

#### *In-vivo* fertility trials

Statistical data showed that 5 μM quercetin was found to be effective in terms of CASA parameters, structural semen parameters, and antioxidant/oxidant indices. Approximately, 60 clinically fit multiparous crossed breed Kamori goats also known as Pateri goats, which are the cross of (Kamori x Gulabi), were assigned in the surrounding area of Sabu Rahu, Sindh, Pakistan. Deworming and vaccination schedules were followed prior to the start of the experiment. Estrus synchronization was performed using two methods: Ovsynch, a fixed-time artificial insemination protocol (50), and medroxyprogesterone acetate (MAP) sponges (51, 52). To rectify the *in-vivo* fertility results, the MAP sponges group (*n* = 30) was inseminated with 5 μM quercetin, while the Ovsynch group (*n* = 30) was inseminated with non-antioxidant treated semen, serving as the control group. The double insemination was performed with each insemination having 50 × 10^6^ sperms/0.5 mL French straw.

#### Ultrasonography for pregnancy detection

Pregnancy was further confirmed through trans-rectal ultrasound scanning on day 40 post-insemination using an ALOKA ECHO CAMERA Model SSD-500 equipped with a 3.5 MHz linear array transducer. Clinical pregnancy was examined when the gestational sac was observed under trans-rectal ultrasonography.

### Statistical analysis

Data were expressed as mean ± standard error, and the Shapiro–Wilk test was used to check whether the data were normal. Data of different variables were analyzed using one-way analysis of variance (ANOVA), and the Tukey test was performed to compare the means of different groups and Pearson correlation by using SPSS 20.0 software (SPSS Inc., Chicago, IL, USA). The chi-square statistical model was applied for *in-vivo* fertility data. The probability values of < 0.05 were taken to indicate statistical significance.

## Results

### Sperm kinematics parameters

The different values of post-thaw semen parameters using the CASA system are shown in Table 1. Quercetin at 0, 1, 5, 10, and 15 μM significantly improved total motility (*p* < 0.001), progressive motility (*p* < 0.004), and rapid velocity (*p* < 0.001), in contrast to the control group. Similarly, average path velocity (*p* < 0.009), straight line velocity (*p* < 0.038), curvilinear velocity (*p* < 0.001), and lateral head displacement (*p* < 0.047) were significantly higher in the semen groups consisting of 0, 5, 10, and 15 μM quercetin.

**Table 1:** Effect of adding quercetin in semen extender on post-thawed spermatozoa quality parameters.

Parameters	Groups					
	Control	1 μM	5 μM	10 μM	15 μM	*p*-value
TM (%)	43.00 ± 6.93^c^	60.33 ± 3.12^b^	85.33 ± 1.20 ^a^	80.67 ± 1.86 ^a^	85.00 ± 1.00 ^a^	<0.001
PM (%)	22.33 ± 2.11^c^	33.20 ± 1.80^a^	34.14 ± 1.16^a^	28.40 ± 2.11^b^	27.60 ± 1.22^b^	<0.004
RV (%)	25.67 ± 3.45^b^	36.67 ± 1.76^b^	66.33 ± 1.76^a^	56.67 ± 2.96^a^	61.33 ± 1.33^a^	<0.001
VAP (μm/s)	62.33 ± 0.88^b^	62.67 ± 3.48^b^	75.67 ± 3.84^a^	67.00 ± 1.00^ab^	69.67 ± 0.88^ab^	<0.009
VSL (μm/s)	45 ± 0.00^c^	45.67 ± 2.19^c^	55.00 ± 3.61^a^	49.33 ± 1.33^b^	48.67 ± 0.88^b^	<0.038
VCL (μm/s)	91 ± 3.00^c^	95.67 ± 3.76^bc^	119.67 ± 5.24^a^	107.67 ± 1.20^ab^	112.33 ± 2.19^a^	<0.001
ALH (μm/s)	4.0 ± 0.58^a^	5.0 ± 0.00 ^a^	6.0 ± 0.58^ab^	5.67 ± 0.33^ab^	5.67 ± 0.33^b^	<0.047
BCF (Hz)	18.0 ± 0.0	20.0 ± 0.0	20.0 ± 0.58	19.33 ± 0.33	20.67 ± 1.67	0.252
STR (%)	68.33 ± 0.33	70.33 ± 0.88	69.0 ± 1.16	70.67 ± 1.20	67.67 ± 0.88	0.196
LIN (%)	46.67 ± 1.76	48.67 ± 0.33	45.67 ± 0.67	46.00 ± 1.00	43.67 ± 0.88	0.073
ELONG (%)	49.33 ± 1.33	47.00 ± 2.08	46.67 ± 0.33	47.33 ± 1.33	47.00 ± 0.58	0.617

Values are displayed as mean ± standard error, three independent replicates are used for total motility (TM), progressive motility (PM), rapid velocity (RV), average path velocity (VAP), straight line velocity (VSL), curvilinear velocity (VCL), the amplitude of lateral head displacement (ALH), beat cross frequency (BCF), straightness (STR), linearity (LIN), and elongation (ELONG). ^a-c^ Mean values sharing different superscripts differ significantly (*p* < 0.05).

### Acrosomal integrity, plasma membrane integrity, viability, and DNA integrity

The plasma membrane and acrosome activity, the viable percentage of spermatozoa, and the DNA integrity of quercetin-treated spermatozoa are illustrated in Table 2. The membrane integrity was reported to be higher (*p* < 0.001) in the group treated with 5, 10, and 15 μM of quercetin than in other groups. Conversely, significantly increased viable sperm with intact acrosome (V/IACR) (*p* < 0.001) and percentage of viable spermatozoa (*p* < 0.013) were observed in 5, 10, and 15 μM levels of quercetin, as opposed to other groups. All treated groups of quercetin have failed to provide a significant effect (*p* > 0.275) on the DNA integrity of spermatozoa.

**Table 2:** Effect of quercetin on frozen–thawing plasma membrane integrity, viable sperm with intact acrosome, viability, and DNA integrity of spermatozoa.

Parameter (%)	Groups					
	Control	1 μM	5 μM	10 μM	15 μM	*p*-value
PMI	60.86 ± 1.31^c^	63.53 ± 2.31^bc^	70.80 ± 0.85^a^	69.43 ± 0.63^ab^	71.20 ± 1.06^a^	< 0.001
ACR	61.76 ± 0.95^b^	63.16 ± 1.25^b^	69.13 ± 0.46^a^	68.56 ± 0.58^a^	67.93 ± 0.32^a^	< 0.001
Viability	62.96 ± 1.30^b^	65.58 ± 2.89^ab^	71.79 ± 1.77^a^	71.20 ± 1.19^ab^	72.41 ± 1.27^a^	< 0.013
DNA integrity	95.23 ± 1.49	97.06 ± 0.32	97.76 ± 0.43	97.86 ± 0.76	97.46 ± 0.87	0.275

Mean ± Standard error. The values of three independent replicates for plasma membrane integrity (PMI), viable sperm with intact acrosome (V/IACR), and the percentage of live spermatozoa (Live). ^a-c^ Mean values sharing different superscripts differ significantly (*p* < 0.05).

### Antioxidant/oxidant sperm parameters

The results of freeze-thawing oxidant/antioxidant status of Kamori bucks are summarized in Table 3. The SOD, CAT, POD, and APX were increased (*p* < 0.05) with the inclusion of quercetin in treated groups compared to the control group. However, quercetin also significantly reduced (*p* < 0.05) TOS and MDA levels while enhancing TAC levels (*p* < 0.05) in treated groups compared to the control group. Among the treated groups, quercetin at 5 μM was found to be an effective concentration with increased antioxidant and reduced oxidant status.

**Table 3:** Impact of adding quercetin in semen extender on freeze-thawing antioxidant/oxidant status in Kamori bucks.

Parameter	Groups					
	Control	1 μM	5 μM	10 μM	15 μM	*p*-value
SOD (Units/ml)	22.81 ± 0.72^b^	25.74 ± 0.29^b^	30.50 ± 0.50^a^	26.18 ± 0.93^b^	25.50 ± 0.50^b^	< 0.003
CAT (Units/ml)	5.0 ± 0^b^	6.0 ± 1.0^b^	9.50 ± 0.50^a^	9.0 ± 1.0^b^	7.0 ± 0.0^b^	< 0.015
POD (Units/ml)	7.0 ± 0^b^	7.50 ± 0.5^b^	11.0 ± 0^a^	6.5 ± 0.50^b^	7.0 ± 1.0^b^	< 0.004
APX (Units/ml)	7.50 ± 0.50^c^	11.50 ± 0.50^ab^	13.50 ± 0.50^a^	8.50 ± 0.50^c^	9.50 ± 0.50^bc^	< 0.002
TOS (μM/ml)	4915.35 ± 36.65^a^	3959.60 ± 52.40^b^	2,157 ± 57^c^	2354.40 ± 31.40^c^	2158.25 ± 13.25^c^	< 0.001
MDA (μM/ml)	98532.53 ± 7.11^a^	66122.03 ± 4.53^c^	59019.66 ± 5.53^cd^	63821.26 ± 18.41^d^	80826.94 ± 3.37^b^	< 0.001
TAC (μM/ml)	1.0 ± 0.0^c^	1.51 ± 0.0^b^	1.90 ± 0.01^a^	1.73 ± 0.04^a^	1.81 0.03^a^	< 0.001

Mean ± Standard error. Values of three independent replicates for superoxide dismutase (SOD), catalase (CAT), peroxidase (POD), ascorbate peroxidase (APX), total oxidant status (TOS), malondialdehyde (MDA), and total antioxidant capacity (TAC). ^a-d^ Mean values sharing different superscripts differ significantly (*p* < 0.05).

### Pregnancy rate through AI

The results of pregnancy confirmation through artificial insemination are shown in Table 4. Ultrasound results showed that 18 out of 30 (60%) animals and 24 out of 30 (80%) animals from the control group and the quercetin-treated semen group, respectively, were found to be pregnant. The pregnancy rate tended to be higher (*p* > 0.079) for the quercetin-treated semen group compared to the control group.

**Table 4:** Pregnancy confirmation through ultrasound machine.

Treatments	Pregnant (*n*)	Non-pregnant (*n*)	Total (*n*)	Pregnancy (%)	Chi-square	*p*-value
Control	18	12	30	60	0.091	0.079
Quercetin treated	24	06	30	80		
Overall	42	18	60	70		

Values are expressed as numbers and percentages. Chi-square showed non-significant results.

## Discussion

The ability of spermatozoa to assess the sperm kinematics parameters and other sperm structural attributes, such as functional plasma membrane integrity, acrosome integrity, DNA integrity, and viability, are important entities for successful fertilization (53, 54). In our study, total motility, progressive motility, rapid velocity, average path velocity, straight line velocity, curvilinear velocity, and amplitude of lateral head displacement were significantly increased in the semen extender group treated with quercetin compared with other groups. Among all treated groups, quercetin at 5 μM concentration provided the best motility and velocity parameters. Previous studies showed that adding quercetin with other compounds or alone promoted different frozen–thawed sperm motion kinetic parameters in goat semen (55), sheep (56), and bull semen (57). Quercetin is a potent antioxidant compound that acts as a scavenger of reactive oxygen species (RNS) (58). It can preserve frozen–thawed sperm viability, DNA integrity, and functionality in bovine (59), ram (30), and humans (60). Quercetin, when combined with other compounds, increases antioxidant activity, thereby defending the plasma membrane against the detrimental effect of ROS and hence supporting sperm motion kinetic parameters (55).

The plasma membrane and acrosomal integrity are the key components of spermatozoa that play important roles in penetrating the ovum and are responsible for sperm survival. When these two structures are damaged during the cryopreservation process, they cause detrimental effects on sperm cells and eventually compromise sperm survival, resulting in poor fertilizing potential (61). Our study showed that 5, 10, and 15 μM of quercetin produced a beneficial effect on plasma membrane and acrosome integrity. Our results are in line with previous studies that showed improved intactness of the plasma membrane and acrosome integrity due to quercetin in post-thawed Egyptian buffalo bulls (62). Oxidative stress influences membrane integrity and acrosome integrity, while the addition of quercetin in semen ameliorated oxidant production and conferred a protective effect on the plasma membrane and acrosome integrity in jungle fowl (63). The DNA integrity of spermatozoa is a pivotal variable for assessing semen quality and is known to be related to fertility in bull (64), stallion (65), bucks (66), and buffalo (57). We used acridine orange assay for the estimation of DNA integrity; green fluorescence indicates the normal DNA pattern while red fluorescence shows DNA damage (67). In this study, we found non-significant differences between sperm DNA integrity among control and all quercetin-treated groups. Our results are in agreement with previous studies in which egg yolk consisted of ovine and Red, Triladyl®, and Biladyl® in frozen–thawed white-tail deer spermatozoa (68). It is well-established that abundant ROS causes DNA fragmentation during the freeze-thawing process (69). Furthermore, a non-significant effect was observed in sperm DNA damage among the control group and the 0.040 mg/mL quercetin-treated group in post-thaw rooster semen (70).

The viability of spermatozoa is a fertility predictor in which live and dead sperm cells are counted using eosin and nigrosin dyes (71). We found that all levels of quercetin showed a significant effect on sperm viability compared to the control group. The freezing–thawing process reduces sperm viability, normal morphology, and motility but enhances DNA damage (72). Our results corroborated Mahmoud et al.’s study (73), in which *Moringa oleifera* increased sperm quality parameters including the viability of spermatozoa. The addition of organic zinc and copper (antioxidant properties) in the buck’s diet improved sperm viability, plasma membrane and acrosome integrities, and CASA parameters, which were compromised due to oxidative stress (74).

Spermatozoa are naturally embedded with an antioxidant defense that encompasses superoxide dismutase, catalase, glutathione peroxidase, and glutathione reductase (75). Overproduction of ROS significantly impairs the structural and functional intactness of sperm and eventually causes reduced fertility (54). In our study, different levels of quercetin significantly increased SOD, CAT, POD, and APX levels by reducing TOS and MDA levels and thus improved TAC activity, suggesting the protective effects of quercetin against ROS-triggered cryo-damage. Our results are in line with previous studies on rooster and bovine semen in which varying levels of quercetin significantly increased SOD, CAT, and GPx and reduced MDA and ROS concentrations (70, 76). Enzymatic antioxidants, such as SOD, CAT, and GPx, are naturally localized within cells to remove excess ROS [10]. The addition of quercetin increased the levels of SOD, CAT, GSH, and GPx, which can inhibit oxidative stress (70, 77, 78). Quercetin, an antioxidant compound, has shown beneficial effects on fresh and frozen–thawed sperm parameters in different species (20–23). It is involved in neutralizing and suppressing free radical production via interacting with α-tocopherol and eventually declining lipid peroxidation (24). In other studies, quercetin enhanced TAC contents that improved semen parameters in humans and goats (79, 80). Barranco et al. (81) revealed that high contents of TAC in boar seminal plasma were related to sperm survival and fertility and can be used as a marker for predicting fertility.

From our findings, the 5 μM quercetin-treated semen group reported a higher (80%) conception rate than the control (60%) group, showing that both control and treated groups are suitable for artificial insemination in field conditions. Perhaps this favorable effect might be due to the addition of quercetin in the semen extender, optimizing temperature shocks, season, and skills of AI technicians and management of the animals that ultimately improved conception rate. Al-Faruque et al. (82) reported that 39 out of 48 goats conceived using frozen semen through AI. CASA and other semen parameters play a crucial role in determining sperm quality with biological significance during cryopreservation (83) and fertility. Additionally, sperm motility and motion parameters significantly impact both *in-vivo* and *in-vitro* fertilization potential (53). Based on our findings, it can be conferred that cryo-damages were minimal in the 5 μM quercetin treated group compared to the control group. The purpose of our study was fulfilled by quercetin serving as the best option for improving the conception rate in the caprine artificial insemination program. However, further studies are required for using varying levels of quercetin and conducting large-scale *in-vivo* field trials of artificial insemination in goats during different seasons and with semen extenders.

## Conclusion

This study concludes that 5 μM quercetin to semen extender increased antioxidant status and attenuated oxidative stress, which, in turn, improved sperm kinematic parameters and sperm structural variables that ultimately promoted *in-vivo* fertility in crossbred Kamori goats. Further studies are warranted on a large-scale basis *in-vivo* fertility trials in field conditions in the consideration of other factors such as season, management conditions, and parity.

## Data availability statement

The raw data supporting the conclusions of this article will be made available by the authors, without undue reservation.

## Ethics statement

The animal study was approved by the experiment was conducted in accordance with the guidelines of the Institutional Animal Care and Use Committee of the Animal Sciences Division, Nuclear Institute for Agriculture and Biology in Faisalabad, Pakistan, according to number of (NIAB-C-PIEAS-0023). The study was conducted in accordance with the local legislation and institutional requirements.

## Author contributions

IB: Data curation, Methodology, Writing – original draft. MF: Methodology, Validation, Writing – review & editing. AH: Data curation, Formal analysis, Methodology, Writing – review & editing. SA: Supervision, Validation, Writing – review & editing. RK: Supervision, Validation, Writing – review & editing. MS: Investigation, Supervision, Validation, Writing – review & editing. YM: Supervision, Validation, Writing – review & editing. AO: Investigation, Supervision, Validation, Writing – review & editing. GM: Investigation, Methodology, Validation, Writing – review & editing. AD: Formal analysis, Software, Writing – review & editing. TH: Conceptualization, Formal analysis, Funding acquisition, Investigation, Methodology, Project administration, Resources, Software, Supervision, Validation, Visualization, Writing – review & editing.
